# The Impact of Melatonin on Colon Cancer Cells’ Resistance to Doxorubicin in an In Vitro Study

**DOI:** 10.3390/ijms18071396

**Published:** 2017-06-29

**Authors:** Magdalena Fic, Agnieszka Gomulkiewicz, Jedrzej Grzegrzolka, Marzenna Podhorska-Okolow, Maciej Zabel, Piotr Dziegiel, Karolina Jablonska

**Affiliations:** 1 Department of Histology and Embryology, Wroclaw Medical University, Wroclaw 50-368, Poland; fima75@onet.eu (M.F.); agnieszka.gomulkiewicz@umed.wroc.pl (A.G.); jedrzej.grzegrzolka@gmail.com (J.G.); marzenna.podhorska-okolow@umed.wroc.pl (M.P.-O.); maciej.zabel@umed.wroc.pl (M.Z.); piotr.dziegiel@umed.wroc.pl (P.D.); 2 Department of Histology and Embryology, Poznan Medical University, Wroclaw 60-781, Poland; 3 Department of Physiotherapy, University School of Physical Education, Wroclaw 51-612, Poland

**Keywords:** melatonin, P-glycoprotein, multi drug resistance, doxorubicin

## Abstract

Multi-drug resistance (MDR) is the main cause of low effectiveness of cancer chemotherapy. P-glycoprotein (P-gp) is one of the main factors determining MDR. Some studies indicate the potential role of melatonin (MLT) in MDR. In this study, we examined the effect of MLT on colon cancer cell’s resistance to doxorubicin (DOX). Using the sulforhodamine B (SRB), method the effect of tested substances on the survival of LoVo (colon cancer cells sensitive to DOX) and LoVo_DX_ (colon cancer cells resistant to DOX) was rated. Using immunocytochemistry (ICC), the expression of P-gp in the LoVo and LoVo_DX_ was determined. With the real-time PCR (RT-PCR) technique, the *ABCB1* expression in LoVo_DX_ was evaluated. Based on the results, it was found that MLT in some concentrations intensified the cytotoxicity effect of DOX in the LoVo_DX_ cells. In the ICC studies, it was demonstrated that certain concentrations of MLT and DOX cause an increase in the percentage of cells expressing P-gp, which correlates positively with *ABCB1* expression (RT-PCR). The mechanism of overcoming resistance by MLT is probably not only associated with the expression of P-gp. It seems appropriate to carry out further research on the use of MLT as the substance supporting cancer chemotherapy.

## 1. Introduction

Over many years of in vivo and in vitro research, differences in the sensitivity of certain cancer cells to chemotherapy have been observed. This phenomenon is one of the biggest problems of contemporary oncology, and is defined as multidrug resistance (MDR). Based on extensive studies, there are two types of MDR tumor cells. The first group is made up of tumors that originate from tissues with high expression of the transporter proteins, and manifest intrinsic multidrug resistance to cytostatic agents before starting chemotherapy. The second group includes tumor cells that acquire the resistance phenotype during chemotherapy [1]. Depending on the processes leading to resistance, there are two mechanisms of MDR: cellular and non-cellular [2]. The cellular mechanism is further divided into non-classical and classical [1]. Non-classical MDR mechanisms involve enzyme systems that limit the activity of the drug without altering its effective concentration inside the cell. The classical MDR mechanism is conditioned by the membrane transport proteins. Their task is to remove the chemotherapeutic agent from the cell, which reduces its effectiveness by decreasing the intracellular concentration [3]. These proteins belong to the group of ABC (ATP-Binding Cassette) transporters [4]. The best known protein from this group is P-glycoprotein (P-gp) [5].

P-gp is a membrane protein of resistance acting through an ATP-dependent pump [6]. The P-gp substrates include anthracyclines, alkaloids and other natural compounds, as well as certain drugs such as calcium channel blockers, anti-viral protease inhibitors, and various xenobiotics [1,7]. Thus, it is a protein which, on one hand protects cells against toxic substances, while, on the other hand, it reduces the bioavailability of cytostatics, thereby reducing the efficacy of anticancer therapy. P-gp is encoded by the *ABCB1* gene located on chromosome 7 [8]. Expression of P-gp is observed in many normal cells of the body, e.g., in the adrenal glands, in the epithelial cells of renal tubules, as well as in hepatocytes [9]. Overexpression of P-gp is also found in many tumor cells, e.g., acute leukemia, breast and ovarian cancers, resulting in the poor response to chemotherapy compared to the P-gp negative tumors [10,11,12].

Melatonin is a neurohormone synthesized in mammals mainly in the pineal gland. It is also produced by other tissues, including the retina, bone marrow cells and the gastrointestinal tract [13]. The MLT production is associated with circadian rhythm and increased significantly after dark. Melatonin level is high at night and low during the day [14,15]. MLT is an amphipathic substance [16]. So far, three basic mechanisms of MLT action on the cells have been described: through cell membrane receptors (MT1 and MT2) by direct interaction with the cytoplasmic protein or through the nuclear retinoid orphan receptors/retinoid Z receptor β (ROR/RZR) [17,18]. Among the membrane melatonin receptors in mammals, only MT1 and MT2 receptors have been identified. Their activity is associated with activation of G proteins that inhibit adenylate cyclase resulting in lower intracellular levels of cyclic adenosine monophosphate (cAMP), which activates the appropriate signaling pathways [19,20,21,22]. MLT penetrates through cell membranes into cells, where in the cytoplasm, combined with calmodulin, it affects cytoskeletal organization [23,24]. Previous reports have also indicated that MLT interacts with the nuclear orphan receptors from the retinoid-related orphan receptor α/retinoid Z receptor α family participating in of immunological processes, differentiation of the nervous system cells and maturation of T cells [25]. However, recent studies also suggest that RORα is a receptor for the sterol and hydroxyl derivatives of vitamin D, not for melatonin [17,26]. Furthermore, MLT exhibits strong antioxidant properties and affects the increased expression of antioxidant enzymes [27]. MLT exhibits many properties such as antioxidative, oncostatic, antiproliferative, anti-apoptotic, immunostimulatory and has an influence on many metabolic pathways [16,23,28,29].

Doxorubicin (DOX) is a chemotherapeutic agent belonging to the anthracycline I generation, widely used in oncology [30,31]. It is used in the treatment of breast cancer, solid tumors in children, soft tissue sarcomas and aggressive lymphomas [32]. DOX acts mainly through interaction with topoisomerase II (Topo II). The consequence of blocking the function of topoisomerase through the anthracyclines is the fragmentation of DNA, inhibition of cell proliferation and consequently cell death [33]. The cytotoxic effects of DOX action, especially its strong cardio-, nephro- and myelotoxic action, significantly limits its use [34].

There is still ongoing research aimed at finding modulators of P-gp—substances that are able to block the function of P-gp in tumor cells and thereby increase the effectiveness of chemotherapy. It is hypothesized that such a mechanism of action demonstrates MLT [35]. MLT is a substance that may have an influence on the inhibition of cancer cells’ proliferation and may also increase the effects of some cytostatics on these cells [35,36]. On the other hand, it is known that MLT through its antioxidant properties, protects the normal cells of the body from the harmful effects of anticancer drugs [34,37]. However, the question arises as to whether MLT affects the MDR in cancer cells or not. Therefore, the main aim of our work was to evaluate the effect of MLT on colon adenocarcinoma cell’s resistance to doxorubicin (DOX).

## 2. Results

### 2.1. SRB Test

**Figure 1:**
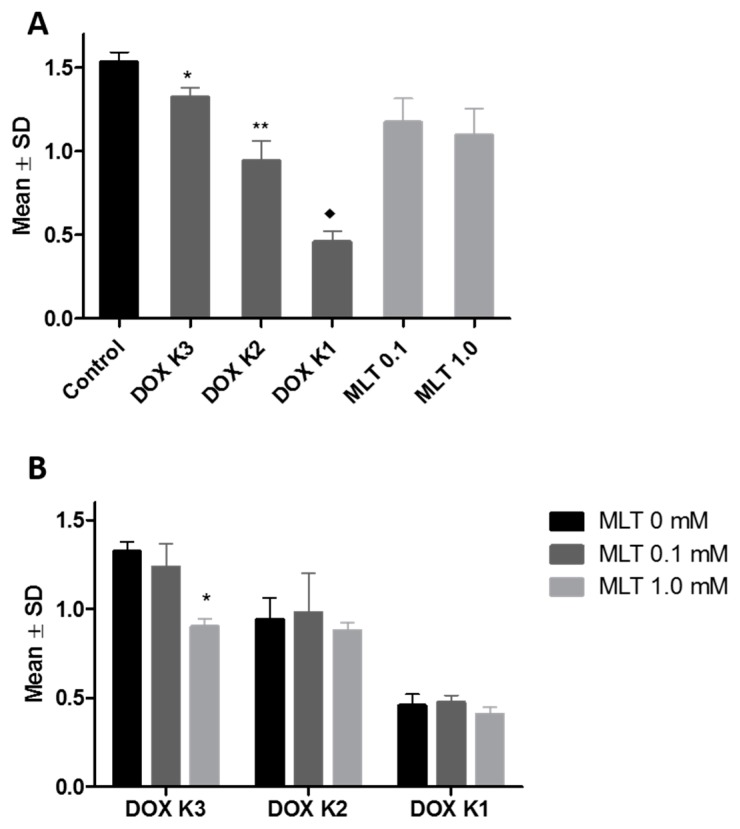
(**A**) The impact of the cytotoxic action of doxorubicin (DOX) and melatonin (MLT) on the LoVo cell line (colon cancer cells sensitive to DOX). The mean optical density (MOD) of LoVo cells decreases with an increasing concentration of DOX: * vs. DOX K1 (0.9 µM) (*p* < 0.01); ** vs. control (*p* < 0.05); ♦ vs. control (*p* < 0.001). MLT had no significant effect on the level of MOD of the LoVo cell line; (**B**) The impact of DOX and MLT combinations on the MOD of the LoVo cell line. The lowest MOD of LoVo cells was observed with a combination of DOX at a concentration of 0.005 mg/mL (0.009 µM, K3) with MLT at a concentration of 1.0 mM: * vs. MLT 0 mM (*p* < 0.01). The data shown are the mean optical density ± SD of three independent experiments.

The SRB test of the LoVo cell line (colon cancer cells sensitive to DOX) demonstrated a statistically significant increase of DOX cytotoxic activity, proportional to the increasing concentration of cytostatic: 0.005 mg/mL (0.009 µM, K3), 0.05 mg/mL (0.09 µM, K2—therapeutic concentration) and 0.5 mg/mL (0.9 µM, K1). IC_50_ for DOX reached 0.69 µM. MLT, at the concentrations of 0.1 mM and 1.0 mM, did not affect significantly the level of mean optical density (MOD) of the LoVo cell line. IC_50_ for MLT reached 2.1 mM (Figure 1A). A significant increase of cytotoxic activity of DOX after the addition of MLT to the culture medium was observed in combination: DOX at a concentration of 0.005 mg/mL (0.009 µM, K3) and MLT at a concentration of 1.0 mM (Figure 1B).

**Figure 2:**
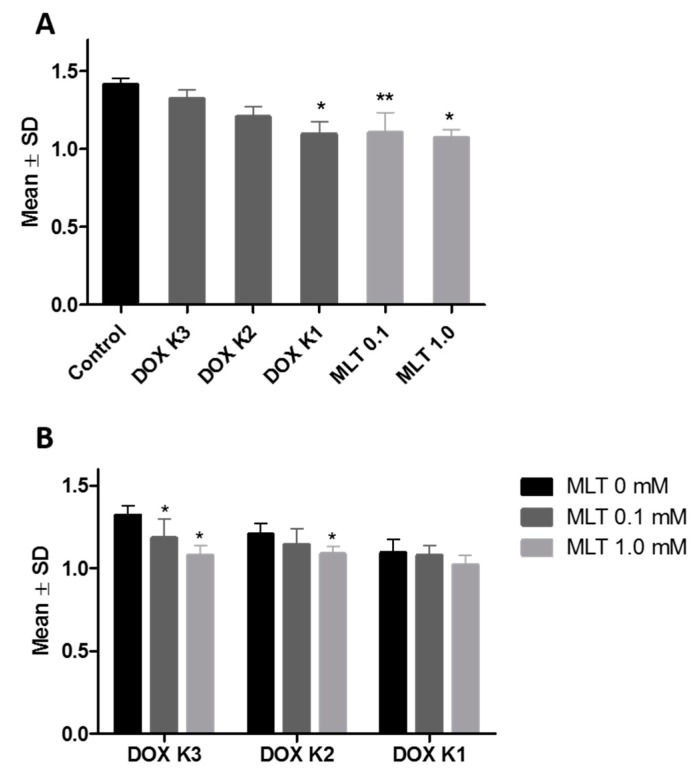
(**A**) The impact of the cytotoxic action of DOX and MLT on the LoVo_DX_ cell line. MOD of LoVo_DX_ is the lowest after incubation with DOX at a concentration of 0.5 mg/mL (0.9 µM, K1): * vs. control (*p* < 0.01); ** vs. control (*p* < 0.05); (**B**) The impact of DOX and MLT combinations on the MOD of the LoVo_DX_ cell line. MLT increased the cytotoxicity of DOX K3 (0.009 µM) at concentrations of 0.1 and 1.0 mM. MLT (1.0 mM) with DOX K2 (0.09 µM) significantly decreased the MOD of LoVo_DX_ cells compared to anthracycline alone: * vs. MLT 0 mM (*p* < 0.05). The data shown are the mean optical density ± SD of three independent experiments.

In the LoVo_DX_ cell line (colon cancer cells resistant to DOX), the cytotoxic activity of DOX was much weaker than in the LoVo cell line. A statistically significant effect of DOX on the level of MOD was observed only at the highest concentration of cytostatic (0.9 µM, K1). MLT at both concentrations (0.1 mM and 1.0 mM) reduced the number of LoVo_DX_ cells, similar to the action of DOX K1 (0.9 µM) (Figure 2A). MLT intensified the cytotoxicity of DOX K3 (0.009 µM) at both concentrations (0.1 and 1.0 mM). MOD values of the above combinations of MLT and DOX K3 (0.009 µM), were similar to that exerted at the concentration of DOX K2 (0.09 µM). A combination of DOX K2 (0.09 µM) with MLT (1.0 mM) significantly decreased the MOD value compared to the anthracycline action alone. The cytotoxicity of DOX K1 (0.9 µM) did not change significantly under the influence of MLT at both concentrations (Figure 2B).

### 2.2. Immunocytochemistry

**Figure 3:**
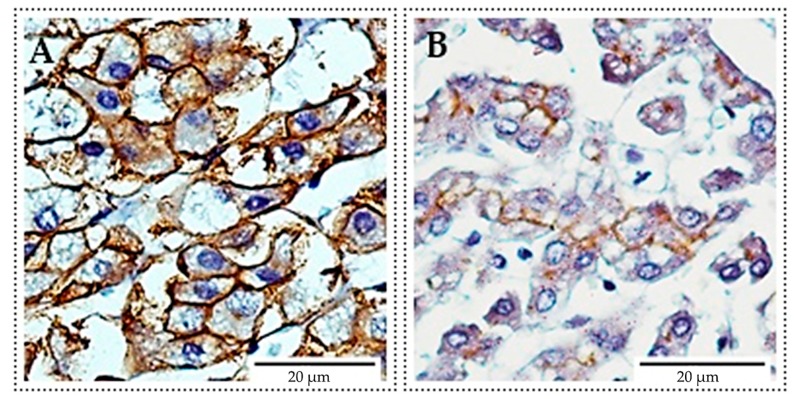
Membrane expression of P-gp (immunohistochemistry (IHC)-positive control). (**A**) Cells of human adrenal glands; (**B**) Cells of human liver. All images were taken at 400× magnification, scale bar corresponds to 20 μm.

In order to demonstrate the membrane location of P-gp protein, the positive control reaction (immunohistochemistry, IHC) was performed on paraffin sections of human adrenal glands and human liver using the anti-P-gp (clone C219) human monoclonal antibody (Figure 3).

**Figure 4:**
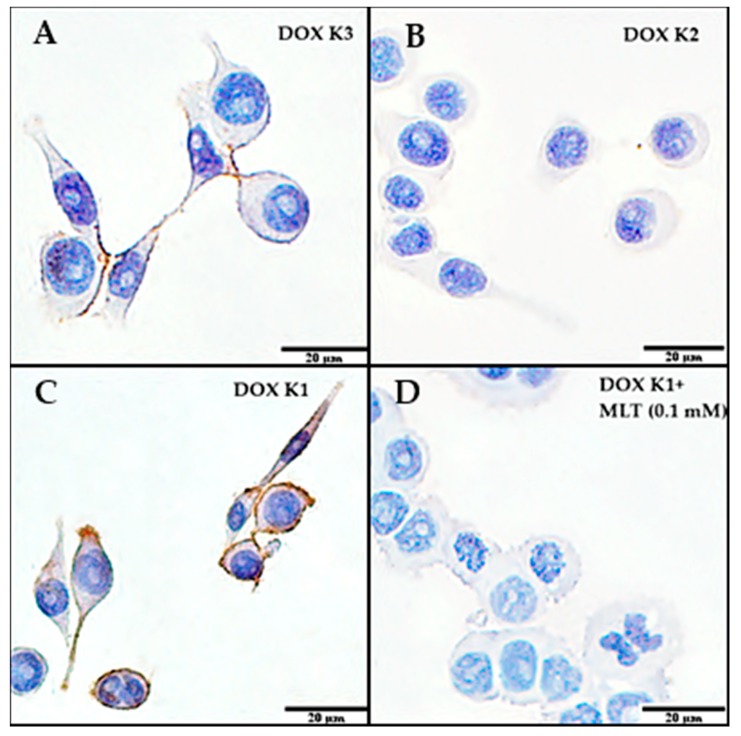
The selected images of immunocytochemistry (ICC) indicate the membrane location of P-gp in the LoVo_DX_ cells with varying intensity of expression depending on the concentrations of DOX and MLT. (**A**) Medium and (**C**) high expression of P-gp; (**B**) Cells treated with DOX at a concentration of K2 (0.09 µM) was characterized by the lowest expression of P-gp; (**D**) Only the addition of DOX K1 (0.9 µM) with MLT (0.1 mM) significantly decreased the percent of cells expressing P-gp in comparison with cells treated with DOX K1 (0.9 µM) alone. Other tested combinations of concentrations of MLT and DOX caused an increase in the percentage of cells expressing P-gp. The images shown are representative of three independent experiments. All images were taken at 600× magnification. Bar corresponds to 20 μm.

Immunocytochemistry (ICC), using monoclonal anti-P-gp antibody, demonstrated the lack of P-gp expression in the LoVo cell lines for all tested combinations of DOX and MLT concentrations. In turn, a strong membrane reaction was observed in some LoVo_DX_ cells treated with various combinations of MLT and DOX (Figure 4A,D).

**Figure 5:**
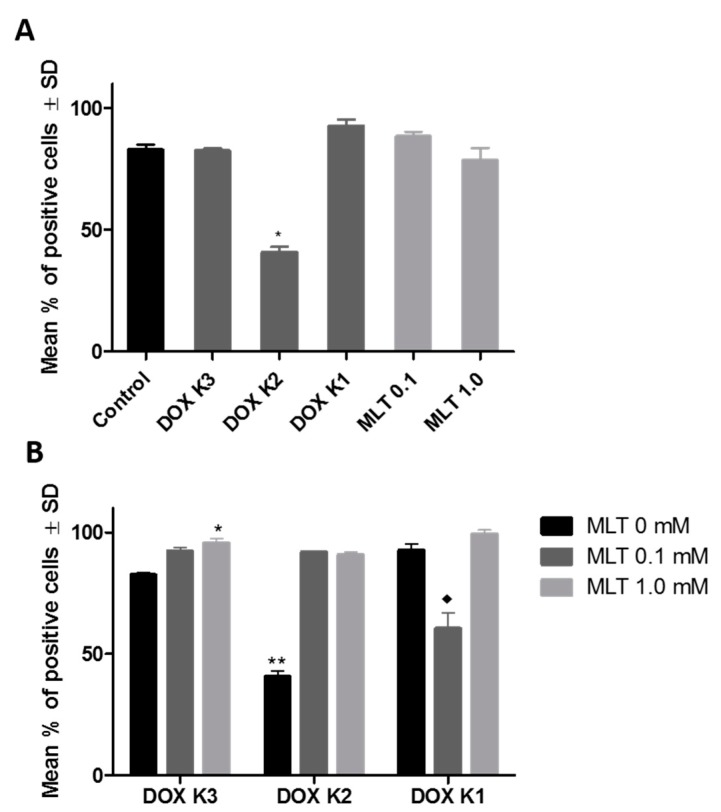
(**A**) The percentage of LoVo_DX_ cells expressing P-glycoprotein in ICC reactions. Only DOX K2 (0.09 µM) significantly decreased the percentage of cells expressing P-gp: * vs. control, (*p* < 0.01); (**B**) The effect of combinations of different concentrations of DOX and MLT on the percentage of LoVo_DX_ cells expressing P-gp in the ICC reaction. Only MLT at a concentration of 0.1 mM with DOX K1 (0.9 µM) significantly decreased the percentage of cells expressing P-gp: * vs. MLT 0 mM (*p* < 0.05); ** vs. MLT 0.1 mM and MLT 1.0 mM (*p* < 0.01); ♦ vs. MLT 0 mM and MLT 1.0 mM (*p* < 0.005). The data shown are the mean % of positive cells ± SD of three independent experiments.

In this experiment, MLT alone did not significantly affect the percentage of LoVo_DX_ cells expressing P-gp. The percentage of cells expressing P-gp was significantly decreased by the action of DOX K2 (0.09 µM) and was 40.69% of all cells (Figure 5A). It was also a low-intensity reaction on the semi-quantitative Immunoreactive Remmele Score (IRS) scale (2 pts acc. Remelle scale [38]). MLT at a concentration of 1.0 mM in combination with DOX K3 (0.009 µM) significantly increased the number of cells expressing P-gp. Additionally, MLT at both concentrations (0.1 and 1.0 mM) significantly increased the percentage of cells expressing P-gp in the case of DOX K2 (0.09 µM), in comparison with cytostatic acting alone. MLT only at a concentration of 0.1 mM significantly decreased the percentage of cells expressing P-gp in the case of DOX K1 (0.9 µM) (Figure 5B). The highest intensity of ICC reaction was observed for LoVo_DX_ control cells and for DOX K1 and K3 doses (12 pts acc. Remelle scale). After application of MLT alone and in the combinations with DOX (K1, K2, K3), the intensity of the reaction was moderate (6–8 pts acc. Remelle scale). No reaction was observed in LoVo control cells (0 pts acc. Remelle scale).

### 2.3. Real-Time PCR

**Figure 6:**
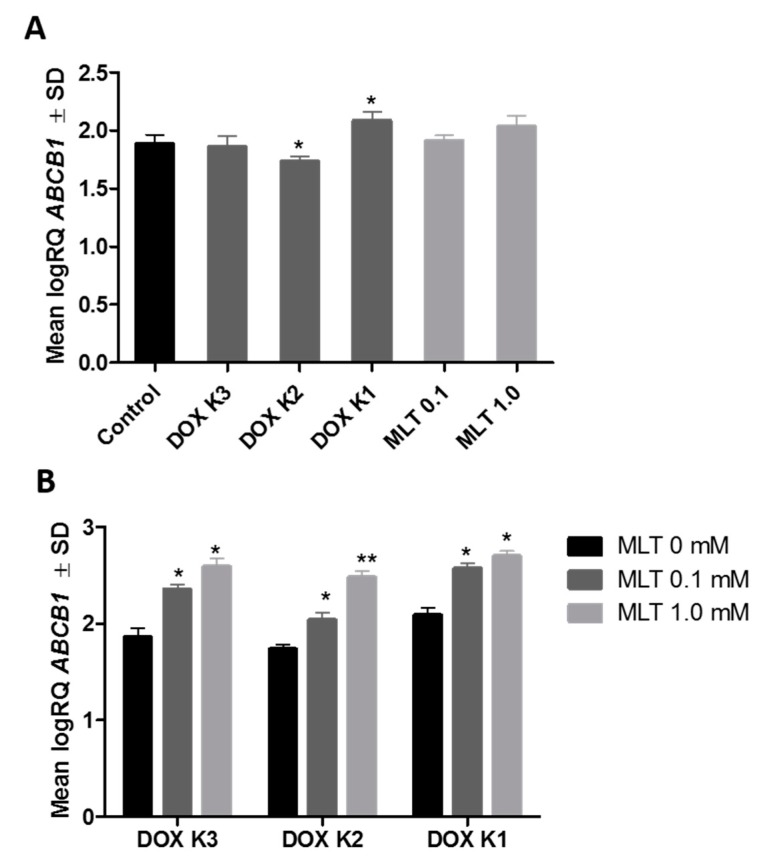
(**A**) Expression of mRNA for P-gp in the LoVo_DX_ cell line after incubation with different concentrations of DOX and MLT analyzed by real-time PCR. MLT alone had no statistically significant influence on *ABCB1* gene expression in LoVo_DX_ cells. DOX K2 (0.09 µM) significantly decreased, while DOX K1 (0.9 µM) significantly increased *ABCB1* gene expression: * vs. control (*p* < 0.01); (**B**) Expression of mRNA for P-gp in the LoVo_DX_ cell line after incubation with various combinations of concentrations of DOX and MLT analyzed by real-time PCR. All combinations of MLT and DOX significantly increased the expression level of the ABCB1 gene: * vs. MLT 0 mM (*p* < 0.01); ** vs. MLT 0 mM and MLT 0.1 mM (*p* < 0.01). The data shown are the mean logRQ ± SD of five independent experiments.

Using real-time PCR, we showed that MLT alone does not significantly affect the expression of the *ABCB1* gene in LoVo_DX_ cells. DOX at a concentration of K2 (0.09 µM) significantly decreased, whereas, at the concentration of K1 (0.9 µM), DOX significantly increased *ABCB1* gene expression (Figure 6A). Combining MLT with DOX at all tested concentrations to the LoVo_DX_ cell culture potentiated the gene expression of P-gp compared to cells treated only with the cytostatic (Figure 6B).

**Figure 7:**
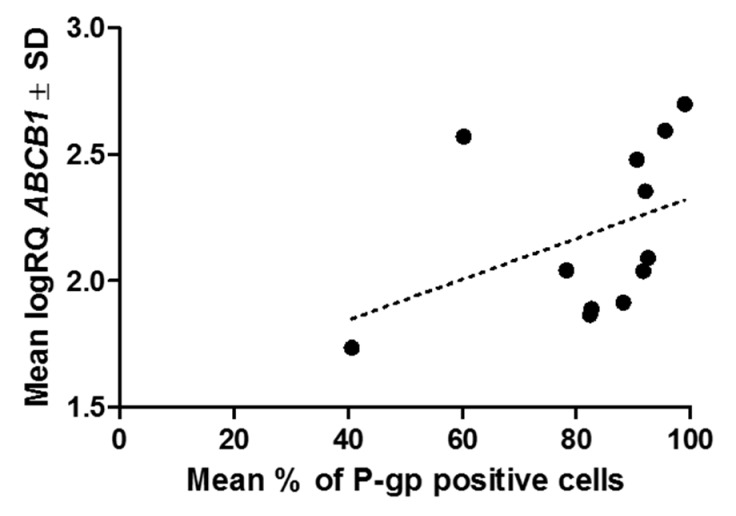
*ABCB1* gene expression (RT-PCR) correlated positively with the percentage of LoVo_DX_ cells expressing P-pg (ICC) (correlation coefficient *r* = 0.42; *p* < 0.05).

In order to confirm the relation between the level of the *ABCB1* gene expression and the P-gp protein expression level (ICC) in the LoVo_DX_ cell line, we found the Spearman’s rank correlation coefficient. The average positive statistically significant correlation between the above variables (correlation coefficient *r* = 0.42; *p* < 0.05) was calculated (Figure 7).

## 3. Discussion

Melatonin is one of the most interesting endogenous substances synthesized in mammals, but the mechanism of its action has not been fully clarified. There are numerous reports indicating the protective properties of MLT against cell damage caused by drugs generating free radicals [32,34,39,40]. MLT (as an adjuvant agent) can be considered to be a substance that may protect cells from the side effects of cancer therapy (both chemo- and radiotherapy). Many in vitro studies have demonstrated the anti-proliferative activity of MLT on tumor cells, including breast cancer—MCF-7, melanoma and colon adenocarcinoma—LoVo [41,42,43,44,45]. The impact of MLT on neoplastic processes is associated with both the receptor and receptor-independent mechanisms [16,46,47].

In our studies, we used MLT at therapeutic concentrations because it seems that its physiological concentrations do not sufficiently protect cells from the toxic effects of anticancer drugs. Even a “megadose” of MLT does not give undesired side effects [34,39,40,48]. In our studies, we confirmed reduced survival of the LoVo and LoVo_DX_ (statistically significant) cell lines under the therapeutic concentrations of MLT. However, we found no discrepancies in the anti-proliferative effect of MLT related to the concentrations of this neurohormone (0.1 mM and 1.0 mM) that were used. It is noteworthy that reduced survival of the LoVo_DX_ cell line caused by the MLT action, was similar to that exerted by the highest concentration of DOX (K1, 0.9 mM). Granzotto et al. [35] carried out in vitro studies similar to ours. They noted that MLT significantly inhibited the proliferation of LoVo cells at a concentration of 100 pg/mL, whereas, for the DOX resistant cell line (LoVo/ADR), this concentration was 1000 to 2000 pg/mL. The mechanism of such action has not been elucidated.

In our experiment, DOX had a much greater impact on LoVo cells than the neurohormone of the pineal gland. With an increased concentration of the cytostatic, the number of cells in the sensitive cell line decreased. For the resistant cell line, a significant cytotoxic effect of anthracycline was marked only at the concentration of K1 (0.9 µM). The cytotoxic effects of DOX on cells are linked primarily to its ability to directly damage the DNA strand. A weaker action of DOX on the LoVo_DX_ line is caused by the presence of MDR proteins in the cells, including P-gp. Evidence of this, as seen in our research, is a membrane expression of P-gp that is associated with increased expression of the *ABCB1* gene. Membrane localization of the protein of resistance is largely responsible for the decrease of intracellular accumulation of cytostatic and changes its distribution. Molinari et al. in their study found that after administration of DOX to cells sensitive to the cytostatic, anthracycline concentration was the highest in the cell nucleus, and thus in the place of its primary activity [49]. However, in the resistant cells, the cell nucleus was completely free of DOX, and the anthracycline was localized exclusively in the cytoplasm. Disorder of cytostatic distribution within the intracellular compartments also affects the effectiveness of the drug. The location of P-gp in the field of the nuclear envelope and in the membranes of intracellular organelles (e.g., the Golgi apparatus), despite an adequate intracellular concentration of DOX, makes the drug ineffective. This phenomenon is associated with insufficient levels of the chemotherapeutic agent at the site of action, i.e., in the cell nucleus [49].

In one of the few publications describing the impact of the simultaneous action of MLT and DOX on tumor cells in vitro, Granzotto et al. found that MLT, to a small degree, not statistically significant, intensified the cytotoxicity effect of DOX at a concentration of IC50 in the LoVo cell line [35]. Similarly, in our studies, only MLT at a concentration of 1.0 mM significantly potentiated the cytotoxicity of DOX K3 (0.009 µM) in the sensitive cell line. With other combinations of drugs, there were no statistically significant differences. In the experiments carried out on the lines HEp-2 and A-549, MLT also potentiated the cytotoxicity of DOX, reduced the number of viable cells, and acted pro-apoptotic [36].

Our studies showed that the MLT at certain concentrations intensified also the cytotoxicity of DOX in the LoVo_DX_ cell line. It is noteworthy that the addition of MLT at a concentration of 1.0 mM to DOX K2 (0.09 µM) caused a decrease in the number of LoVo_DX_ cells to the value that cytostatic alone in the largest used concentration exerts on these cells. This effect, as in our experiment, depended on the concentration of the MLT. According to Granzotto et al., MLT slightly, but statistically significantly, potentiated the cytotoxicity of DOX in LoVo/ADR cells [35]. However, its mechanism is not clear.

MLT has a varying effect on the level of P-gp depending on the dose used, both at the mRNA level and at the number of cells with P-gp expression. In the case of MLT alone, we observed no statistically significant differences in the number of cells expressing P-gp as well as we observed no significant changes in gene expression. In most cases, the addition of MLT and DOX combination was related with an increased percentage of cells expressing P-gp and with increased level of *ABCB1* (statistically significant) in relation to DOX alone. Only addition of the combination of MLT 0.1 mM with DOX K1 to cell culture resulted in a significant decrease in percentage of cells with P-gp expression. Additionally, we noted that after addition of the DOX alone, intensity of color reaction was high on the IRS scale, whereas, after addition of MLT, the intensity of color reaction changed to moderate.

In some publications, it has been suggested that the resistance of tumor cells to cytotoxic drugs is overcome by the MLT influence on the expression or function of P-gp [35]. Our results do not fully support this theory. By real-time PCR, we clearly demonstrated that the addition of MLT to each concentration of DOX significantly potentiates the *ABCB1* gene expression. Some of our ICC results confirmed these observations. Enhanced expression of P-gp examined by ICC in the resistant cell line was the lowest after the addition of DOX at the therapeutic concentration (K2, 0.09 µM), which correlated with a significant decrease of *ABCB1* gene expression. A tenfold increase in the concentration of the chemotherapeutic agent in DOX resistant cells, caused a small, but statistically significant, increase of *ABCB1* gene expression. This suggests that perhaps DOX alone at an appropriate concentration is able to inhibit or potentiate the gene expression of the resistance protein in the LoVo_DX_ cell line. The mechanism of such action is not clear. This is probably related to the theory of Takeshita et al. on the phenomenon of overexpression of the proteins of resistance in more differentiated cells [50].

Takeshita et al., who conducted studies on osteosarcoma cells sensitive and resistant to cytotoxic drugs, suggested that overexpression of P-gp in resistant cells may be linked to their functional differentiation [50]. They hypothesized that the functional differentiation of resistant cells may be reflected in the differentiation of cell morphology, especially in the structure of the microfilaments. Actin filaments of osteosarcoma cells sensitive to cytotoxic drugs are distributed throughout the cytoplasm, whereas the resistant cells manifest a much more ordered arrangement of microfilaments (in the form of thick tufts as in normal cells). In turn, the increased differentiation of cancer cells results in the reduction of their malignancy potential. These findings are confirmed by other studies on protein expression of resistance, in which the administration of agents stimulating cell differentiation resulted in increased expression of P-gp [50]. Furthermore, the overexpression of P-gp in the cells of more differentiated cancers in organs such as the kidney and colon has been observed [51,52,53,54]. It is therefore likely that MLT, as a stimulant of cell differentiation, in the presence of DOX, at some concentrations, may indirectly increase the expression of mRNA of the *ABCB1* gene in LoVo_DX_ cells. This is also confirmed by our results.

There are reports that, in the cells with MDR, high expression of membrane P-gp is not always associated with the high activity of this protein [1]. Modulation of MDR phenomenon in P-gp may rely on the inhibition of the post-translational modification of this protein like acetylation and phosphorylation [55,56,57]. It has been shown that substitution of protein kinase A (PKA) and protein kinase C (PKC) phosphorylable residues in the P-gp linker region affects the stimulation of P-gp ATP-ase activity by some substrates [56]. Inhibition of PKC-α by the p53 decreases the phosphorylation of P-gp in tumor cells of soft tissues, which sensitizes these cells to chemotherapy [57]. It is possible that MLT, indirectly blocking a post-translational modification of P-gp, inhibits its transport function. Baldwin et al. suggests that an oncostatic action of MLT is the result of its interactions with cell membranes and nuclear receptors [51]. Binding of MLT to the MT1 membrane receptor decreases cAMP synthesis via adenyl cyclase inhibition, reduction activity of the protein kinase C (PKC), protein kinase A (PKA), and mitogen-activated protein kinases (MAPK) [24,58]. This mechanism has a negative impact on the phosphorylation of the transcription factor CREB (cAMP response element-binding) and on the expression of the genes involved in proliferation, angiogenesis, and migration processes in breast cancer cells [58,59]. Moreover, the activity of P-gp may also be modulated by the phase of the cell cycle. Takeshita et al. observed that, during mitosis, P-gp function is inhibited, which may be caused by dispersion of actin filaments [50]. However, given that MLT arrests cells in the G0/G1 phase, such a mechanism of hormone action seems to be less likely.

Substrate specificity has a significant effect on P-gp activity. Today, it is known that within the P-gp there are two binding sites that exhibit the ability to bind compounds of different structures and one site with modular functions. It is interesting that one substrate can target both binding sites and regulate the transport of another substrate. It can be assumed that this effect is related to the cooperation of MLT and DOX. Studies conducted by Ferreiras and Loo about molecular docking of substrate characterize the structure of individual binding sites on P-gp [58,59,60]. The P-gp has two to four substrate binding sites. Two different substrates may be bonded at the same time, and different binding sites may attach the same substrates or may be allosteric interrelated [59,61]. Presumably, one of the used substrate (MLT or DOX) might block the entry to the drug-binding pocket, alter the confirmation of P-gp and disturb transport cycle [61]. Another important issue is the availability of drug-binding pocket to aqueous medium. Hydration of the drug after its expulsion to the extracellular matrix may prevent potential diffusion back into the lipid bilayer [62]. Considering this fact, it can be assumed that the ethanol in which MLT was dissolved could have an effect on the reduction of cytostatic activity. It might be an explanation as to why certain concentrations of MLT and DOX caused an increased percentage of cells expressing P-gp.

Results of the newest studies indicate that both ATP binding and ATP hydrolysis by P-gp are modulated by the biophysical state of the lipid bilayer. Lipids can induce structural changes in P-gp and regulate the activation of P-gp ATPase activity. It is interesting that MLT affects the dynamics of membranes in different ways, at different concentrations. Low concentrations of MLT increases fluidity of lipids, while, at high concentrations, it has the opposite effect. At low MLT concentrations, small melatonin-enriched domains with smaller membrane thicknesses while at a high MLT concentrations, a uniform distribution of melatonin throughout the membrane was observed, where the MLT molecules align parallel to the membranes. The results of two-dimensional X-ray diffraction showed that the MLT molecules organize in a crystal-like structure in the membrane plane, where one MLT molecule associates with two lipid molecules. Conformational changes of P-gp can have a significant effect on the drug transport and drug stimulated ATP-ase activity. Understanding the interaction and organization of melatonin in biological membranes is an essential step to explain the relation between melatonin action and activity of P-gp [63].

Recently, systematic screens of the *ABCB1* gene have identified multiple single nucleotide polymorphisms. *ABCB1* polymorphisms appear to be associated with altered P-glycoprotein transporter function, tissue expression, drug disposition, treatment outcome and disease risk [54]. It was reported that, in the treatment of childhood acute lymphoblastic leukemia (ALL), 3435 TT (two copies of the “T” allele) or CT (one copy of “C” and “T” allele) genotypes were associated with lower P-glycoprotein levels than in the CC genotype, resulting in a better permeability of substrates for P-gp (e.g., doxorubicin, epirubicin, vincristine) [54]. It can be assumed that our cell model of LoVo_DX_, which developed and kept resistance to DOX as well as the combinations of cytostatic, which we used with MLT, may have led to some mutations of the *ABCB1* gene and consequently altered the expression of P-gp.

Based on the reports of other authors, we can suppose that co-operation of MLT with DOX may not only be related to P-gp regulation, but may be the result of interaction of many signal pathways. The recent data show that melatonin inhibit endthelin-1 (ET-1) expression and secretion in colon cancer cells. Inhibition of ET-1 is related with an inactivation of transcriptional factors like FoxO1-1 and NF-κβ and, as a result, affects the tumor growth and progression [64].

Based on the obtained results, it was found that some combinations of concentrations of MLT and DOX increased the sensitivity of LoVo_DX_ cells to the cytostatic. The ICC studies demonstrated that certain concentrations of MLT and DOX caused an increased percentage of cells expressing P-gp, which correlated positively with *ABCB1* expression (RT-PCR). Only MLT at a concentration of 0.1 mM with DOX K1 (0.9 µM) significantly decreased the percentage of cells expressing P-gp. It seems that the mechanism of overcoming resistance by MLT is associated not only with the expression of P-gp. Probably, the most likely mechanism of MLT action on cells resistant to DOX is connected with its effect on the metabolic pathways via specific receptors. It is also possible that MLT may have an impact on post-translational modification of the P-gp protein and cytoskeletal proteins. There are still many unknowns about the MLT action on tumor cells. Confirmation in in vitro and in vivo studies of any of the above hypotheses on the role of MLT in overcoming MDR in cancer cells can be useful in cancer therapy.

## 4. Materials and Methods

### 4.1. Cell Culture

LoVo and LoVo_DX_ cells were cultured in vitro in Minimum Essential Medium (MEM; GIBCO, Scotland, UK) supplemented with 10% fetal bovine serum (Lonza, Allendale, NJ, USA), 100 IU/mL penicillin (Sigma, Munich, Germany) and 100 μg/mL streptomycin (Sigma, Munich, Germany). Cells were incubated on 96-well plates for 24 h at 37 °C in the presence of 5% CO_2_. In the studies, melatonin (Sigma, Munich, Germany) and doxorubicin solution (Adriablastina^®^ 2 mg/ml; Pharmacia, Italy) were used. MLT was dissolved in ethanol and a stock solution was prepared in the culture medium. The maximum concentration of ethanol in the wells did not exceed 0.4%. For the LoVo_DX_ line, with each culture medium change, the DOX at a concentration of 0.5 mg/mL was added to the culture medium in order to maintain the resistance of cells to the cytostatic.

### 4.2. Cytotoxicity Assay of Melatonin (MLT) and Doxorubicin (DOX)

MLT solution was added to individual wells to obtain final MLT concentrations of 0.1 mM or 1.0 mM [65,66]. Incubation was continued for 20 min. The wells were then charged with DOX solutions. Final concentrations of DOX in the wells were: 0.005 µg/mL (0.009 µM, K3), 0.05 µg/mL (0.09 µM, K2–therapeutic concentration) or 0.5 µg/mL (0.9 µM, K1) [65,66]. Controls contained cells suspended in medium. Following a consecutive 48 h of incubation, the contents of each well were stained using the SRB technique [67] and values of mean optical density (MOD) were determined using an automatic plate reader, Elx 800 (Bio-Tek Instruments, Winooski, VT, USA), at a wavelength of 562 nm. The SRB method estimates the endogenous protein contents in viable cells [67]. Experiments were repeated three times. The IC50 (inhibitor concentration at 50%) for DOX and MLT was also determined.

### 4.3. Immunocytochemical (ICC) Detection of P-gp

Expression of P-gp in the examined cell lines was assessed by immunocytochemistry using the LSAB+/HRP (labbeled streptavidin biotin/horseradish peroxidase) System kit (Dako Cytomation, Glostrup, Denmark) and mouse monoclonal anti-human P-gp antibody (clone C-219), (Alexis Biochemicals, Lausen, Switzerland) [68]. In order to obtain a positive control, immunohistochemical reaction (IHC) was performed on paraffin sections of the human adrenal gland and human liver from the archival collection of the Histology Department of Wroclaw Medical University. The results of IHC and ICC reaction were evaluated with a light microscope (Olympus BX41, Tokyo, Japan) at 400× and 600× magnification, using a program for computer image analysis (Analysis 3.2, Soft Imagine System GmBH, Münster, Germany). To assess the ICC reactions, the mean values of the P-gp expression of 5 representative fields of view (“hot spots”) for each combination of MLT and DOX were used. In this evaluation, the number of cells expressing P-gp relative to all cells in the “hot spot” was taken into account. The intensity of p-GP expression has been evaluated by IRS (Immunoreactive Score) acc. Remmele [38]. The scale takes into account the percentage of cells with a noticeable reaction (A) and intensity of the reaction color (B). The final score represents the sum of the two values, ranging within the scope of 0–12 (A × B) (Table 1).

### 4.4. Real-Time PCR (RT-PCR) for ABCB1 mRNA Expression Detection

Using RT-PCR, the P-gp (*ABCB1*) gene expression level was evaluated in LoVo_DX_ cells cultured under the different concentrations of DOX and MLT. For the RNA isolation, the RNeasy Mini Kit was used (Qiagen, Germany). The reverse transcription reaction was performed using an Omniscript RT Kit (Qiagen, Germany) and oligo-dT starters (Institute of Biochemistry and Biophysics, Warsaw, Poland). Changes in the expression level of P-gp (*ABCB1*; TaqMan Gene Expression Assay, Applied Biosystems, Waltham, MA, USA) were tested with using 7900HT Fast Real-Time PCR System (Applied Biosystems). The method of relative quantification (RQ) was used. Analysis of the *ABCB1* gene expression was carried out using the RQ Manager 1.2 software (Applied Biosystems, Waltham, MA, USA). The results were standardized, based on expression of the reference gene of β-actin (ACTB; TaqMan Gene Expression Assay, Applied Biosystems, Waltham, MA, USA) [69]. The changes in the level of *ABCB1* gene expression in LoVo_DX_ cells were tested in relation to LoVo cells. The *ABCB1* gene expression test by real-time PCR was repeated five times for the entire experiment. The obtained results were shown in the graphs on a logarithmic scale and subjected to statistical analysis.

### 4.5. Statistical Analysis

The results underwent statistical analysis using Statistica 7.1 software (StatSoft, Krakow, Poland). All experiments were performed at least three times. Data collected were averaged and expressed as mean ± standard deviation (SD). To evaluate the differences between the results of various groups, the following tests and statistical analysis were used: U Mann–Whitney non-parametric equivalent of the Student’s *t*-test; Kruskal–Wallis non-parametric equivalent of the univariate analysis of variance; and evaluation of Spearman’s rank correlation coefficient.

## Figures and Tables

**Table 1:** Expression level of P-gp was evaluated in the scale of 0–12 points (A × B).

A Percentage of Positive Cells Points	Points	B Intensity of Color Reaction	Points
No positive cells	0	No color reaction	0
<10% positive cells	1	Low color intensity	1
11–50% positive cells	2	Reaction color of moderate intensity	2
51–80% positive cells	3	Intense reaction color	3
>80% positive cells	4		
